# Shape Memory Materials from Rubbers

**DOI:** 10.3390/ma14237216

**Published:** 2021-11-26

**Authors:** Arunima Reghunadhan, Keloth Paduvilan Jibin, Abitha Vayyaprontavida Kaliyathan, Prajitha Velayudhan, Michał Strankowski, Sabu Thomas

**Affiliations:** 1 Postgraduate Department of Chemistry, Milad-E-Sherif Memorial College, Kayamkulam, Alappuzha 690502, India; arunimarenjith02@gmail.com; 2 School of Chemical Sciences, Mahatma Gandhi University, Kottayam 686560, India; jibinkp999@gmail.com (K.P.J.); abithavk@gmail.com (A.V.K.); prajipravi.11@gmail.com (P.V.); sabuthomas@mgu.ac.in (S.T.); 3 Polymer Technology Department, Chemical Faculty, Gdańsk University of Technology, 80-233 Gdańsk, Poland; 4 School of Energy Materials, Mahatma Gandhi University, Kottayam 686560, India; 5 International and Interuniversity Centre for Nanoscience and Nanotechnology, Mahatma Gandhi University, Kottayam 686560, India

**Keywords:** shape memory, elastomer, fixity, recovery, applications

## Abstract

Smart materials are much discussed in the current research scenario. The shape memory effect is one of the most fascinating occurrences in smart materials, both in terms of the phenomenon and its applications. Many metal alloys and polymers exhibit the shape memory effect (SME). Shape memory properties of elastomers, such as rubbers, polyurethanes, and other elastomers, are discussed in depth in this paper. The theory, factors impacting, and key uses of SME elastomers are all covered in this article. SME has been observed in a variety of elastomers and composites. Shape fixity and recovery rate are normally analysed through thermomechanical cycle studies to understand the effectiveness of SMEs. Polymer properties such as chain length, and the inclusion of fillers, such as clays, nanoparticles, and second phase polymers, will have a direct influence on the shape memory effect. The article discusses these aspects in a simple and concise manner.

## 1. Introduction to Rubbers and Properties

The first material, known as caoutchouc, is obtained from the weeping tree. This is polyisoprene recovered from the sap of *Hevea Brasiliensis* and is known as natural rubber (NR) in comparing with synthetically produced rubbers. In the course of developing synthetic analogues of NR, similar compounds were found, which can also be cross-linked with sulphur. However, macromolecular compounds, as they have unsaturation, can be cross-linked with sulphur. This unsaturation normally comes from (partly or totally) diene monomers, for example, polyisoprene (synthetic), polybutadiene, styrene–butadiene, or acrylonitrile–butadiene copolymers [1,2,3,4].

Over the years, the importance of rubber to modern life has constantly increased. About one-third of the total global rubber usage is natural rubber (NR); the remaining two-thirds of required rubber is produced synthetically by a great number of industrial countries, well distributed throughout the world. More than half of the world’s production of natural and synthetic rubber is used in tyres and the remainder is for a great variety of industrial and consumer products [5,6].

The most important property of rubbers is elastic behaviour after deformation (either in compression or tension). It is possible to stretch a rubber sample ten times its original length and after removal of the tension, the given sample will return to its original shape and length. In addition, elastomers have many other useful properties under static and dynamic conditions, such as abrasion resistance, impermeability to air and water, and resistance to swelling in oils/solvents, etc. These properties are exhibited at higher, ambient, or lower temperatures and are mostly retained in different climatic conditions and even in ozone rich atmospheres. Rubbers are capable of adhering textile to fabric and metals, which helps in increasing properties such as tensile strength. These composites increase the range of applications of rubber, for example, metal bonded rubber articles have the elasticity of rubber and rigidity of metal. Properties required for rubber depend on the compounding and type of rubber. Desirable properties of rubber may be obtained by using proper chemical additives, process aids, and subsequent vulcanization. The degree of vulcanization is also important, because for a given compound to have a different state of cure there will be a large variation in properties concerning hardness, elasticity, and strength, but typical properties like oil/gasoline resistance and ageing may remain unaltered. Rubbers can form cross-linked structures; these may be long-chain molecules forming coils that can be extended when subjected to even small stresses. These chain segments are flexible and undergo micro Brownian motion at normal temperatures. The rubber molecules assume statistically ordered confirmation when tensile stresses are applied; on the removal of the stress they return to their statistically random confirmation. This ability of rubber to retain a memory of its original unstressed state and return to its original dimension when external forces are removed is utilized in many shape memory applications and will be discussed in detail in this review [7,8].

## 2. An Overview of Shape Memory Effect

The shape memory effect (SME), as the term implies, is often the recovery or regaining of the shape or size of a material as a result of heating it above a particular characteristic temperature. The definitions may vary, but the effect has the same idea [9,10,11]. The materials exhibiting shape memory effect can be categorized under alloys or polymers, the formed being first proposed in the 1980s and studied in detail [10,12,13,14,15,16]. Nitinol, which is an alloy of nickel and titanium, was the foremost studied material in understanding shape memory behaviour and its plausible applications. This material opened up a window to a new era of engineering material, which continues its development [17]. Presently, SME and these materials have been employed in many advanced research areas.

Shape memory polymers (SMPs), after smart materials, are very versatile in their properties and applications. This effect can be exhibited by both virgin polymers and their composites [18,19,20]. Elastomers, such as nitrile butadiene rubber, natural rubber, epoxidized natural rubber, silicon rubber, and polyurethane, etc., are widely studied because of their shape memory effect [21,22,23,24,25,26,27,28,29]. Various fillers, such as silica, graphene oxide, carbon nanotubes, and nano clay, etc., improve the shape recovery, mechanical strength, and elastic modulus, as well as reduce recovery time. The layered fillers improve the thermomechanical properties of the elastomers because of their high surface area, which improves the matrix filler interaction [22,30,31].

**Figure 1:**
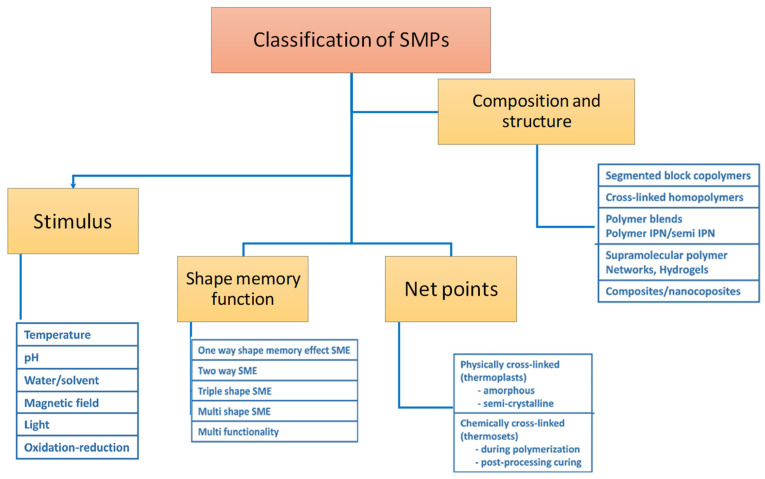
Classification of shape memory polymers, according to different parameters.

The most pronounced stimuli to induce the shape recovery in polymers are electricity, temperature, light, chemicals, and mechanical force. Based on these stimuli, the materials can be further be classified as photoresponsive, thermoresponsive, and photochromic, etc. Shape memory polymers (SMPs) have classifications based on the forces of interaction and polymers involved, and shape memory pattern. A detailed list of categories of SMPs is given in the chart below (Figure 1).

### 2.1. Mechanism behind the Shape Memory Effect in Shape Memory Alloys

**Figure 2:**
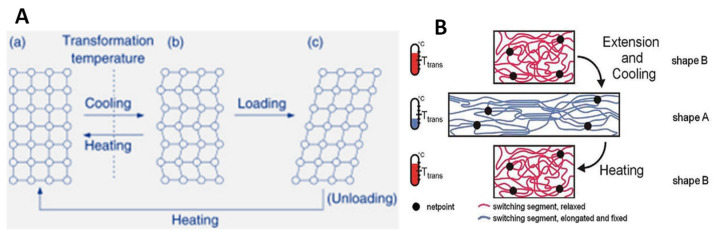
(**A**) Changes in crystalline structure during shape memory effect of alloys and (**B**) mechanism of shape memory in polymers. (Reproduced with permission from [18,33], Elsevier, 2009).

SME cannot be treated as an intrinsic property; it arises as a result of the morphology and the processing conditions of the material. In shape memory alloys, the phase transition between the austenite and martensite crystalline states are responsible for this effect (Figure 2), In shape memory polymers, the rearrangement of the polymeric chains results in the shape memory effect [32]. On the other hand, shape memory changes in polymers are due to the change in the mechanical characteristics above and below the glass transition temperature. Here, we are more concerned about the SME of polymers and, in particular, elastomers. In the case of elastomers, the SME is often combined with other effects, which increase their applications.

Shape memory polymers are flexible polymer networks with appropriate stimuli-responsive switches. The molecular switches and net points make up the polymer network. The net points, which might be chemical or physical in origin, define the permanent form of the polymer network. Physical cross-linking occurs in a polymer with a shape that includes at least two separated regions, such as block copolymers. In this case, the domains corresponding to the greatest transition temperature serve as a net point (hard segment), whilst the chain segments connected to the region with the second-highest thermal transition temperature serve as molecular switches. A temperature that is higher than the transition temperature results in flexible switching domains and an entropy elastic polymer network above the transition temperature limit. It flips back into its original shape if the sample has been previously distorted by external stress when the external tension is released [18,34].In other words, the SME in polymers can be stated as entropy phenomena because its original shape is a thermodynamically stable high entropy state; when it undergoes any macroscopic deformation it changes the entropy and stability. The chains heated above the transition temperature activate mobility in the molecular level; the shape is regained using the energy released from the chains [35,36].

### 2.2. Terms Used in Shape Memory Effect

To explain the phenomena of shape memory we must consider two terms, shape fixity (***R_f_***) and shape recovery rate (***R_r_***). By considering the values obtained for these parameters for a particular material, the efficiency of SMEs can be well understood [37]. Among elastomers, polyurethanes are well known for shape recovery. A great deal of work has been reported on shape memory properties and composites of polyurethanes by Touchy and others [38,39,40,41].The shape fixity can be defined as the switching segment’s capacity to correct or fix transient deformations during the programming procedure. On the other hand, shape recovery (***R_r_***) can be expressed as the shape memory materials’ capacity to recover their unalterable (original) form [42,43,44].

The expression used to calculate the shape fixity (***R_f_***) can be given as

(1) $$Shapefixity,R_{f}=\frac{Permanent\;deformation}{Total\;deformation}$$

For the shape recovery, the equation can be given as,

(2) $$Shape\;recovery\;rate,\;R_{r}=\frac{Deformation\;recovered\;inacertain\;cycle}{Total\;deformation\;inonecycle}\times 100\%$$

The shape fixity (***R_f_***) and the shape recovery (***R_r_***), which are mainly related to the mechanical deformation and capability of recovery from the original shape, and ***R_f_*** and ***R_r_***, are also calculated using the equation shown below [30,45].

(3) $$R_{f}\left(\%\right)=\frac{\varepsilon_{u}}{\varepsilon_{m}}\times 100,\;R_{r}\left(\%\right)=\frac{\left(\varepsilon_{m}-\varepsilon_{p}\right)}{\varepsilon_{m}}\times 100$$

$\varepsilon_{u}=\mathrm{Fixed}\;\mathrm{strain}\;\mathrm{after}\;\mathrm{cooling}\;\mathrm{and}\;\mathrm{load}\;\mathrm{removal}$; $\varepsilon_{m}=\mathrm{Maximum}\;\mathrm{strain}\;\mathrm{when}\;\mathrm{loading}$; $\varepsilon_{p}=\mathrm{Recovered}\;\mathrm{strain}\;\mathrm{after}\;\mathrm{reheating}\;\mathrm{the}\;\mathrm{sample}$.

Thermomechanical cycles are normally performed to analyse SMEs. In this procedure, the samples will be heated to a high temperature normally above their characteristic glass transition temperature (T_g_). The applied strain and resultant strain will be fixed to zero. Presently, the polymer of the composite will be deformed to the desired shape; constant stress and strain are ensured while deforming and fixing to the particular shape. The SMPs are then cooled to a temperature below T_g_, causing the chain segments of the materials to reposition themselves. When the tension is entirely released from the polymer, it is considered to be in its temporary form. Programming is another name for this procedure. When the materials are warmed to temperatures above T_g_, the strain is released, and the materials return to their original form; the thermomechanical cycle is complete. For the following cycle, the recovery processes can be repeated [42]. Very high shape fixity and recovery were reported for polymer composites, which includes thermoplastics [46,47], rubbers, polyurethanes [39,48] and biopolymers [49,50,51,52,53].

## 3. Shape Memory Materials of Rubbers

### 3.1. Natural Rubber (NR) Based Materials with Shape Memory

The superiority of NR over synthetic polymers lies on the fact that they are capable of supporting large amounts of stress up to 30 MPa at a strain of more than 1000%.This is due to strain-induced crystallization (SIC) that occurs whenever cross-linked NR is stretched to large elongations. The crystals formed vanish when releasing the stretching force and regain their original amorphous condition. The effect of SIC was investigated by Katz and thermodynamically by Flory. Flory proposed a theory that considers the entropy change of molecular chains due to stretching. The entropy change is a factor that promotes the SIC and the formation of crystals with low surface energy promotes the enhancement of SIC. In a study by Tosaka et al., the surface energies of strain-induced NR crystals were found to be relatively small. The low surface energy crystallites usually exhibit a high melting point exceeding room temperature; this supports the existence of shape memory natural rubber [54].

### 3.2. Lightly Cross-Linked Shape Memory Natural Rubber

Lightly cross-linked NR networks have a cross-link density below 0.4%; the crystals do not disappear after releasing the stretching force but stabilize the network in a highly elongated state, up to 1000%, at room temperature. The recovery of this network can be triggered by heating it above its melting point, which is known as a trigger temperature. The trigger temperature of shape memory natural rubber (SMNR) is adjustable from −20–50 °C [55]. Lightly cross-linked NR, rapidly stretched and kept in this state, does not recover its original state. But when applying a small heat, such as body temperature, to the stretched material it recovers its original state. Thus, lightly cross-linked NR can be programmed below its triggered temperature, and the stretching of semi-crystalline polymer results in partial recoverability. The cross-linking of NR in-between thermoplastics and heat allow the formation of crystals under strain that can withstand the network in the high heat of elongation. A typical formulation for shape memory natural rubber is given in Table 1 [56].

**Figure 3:**
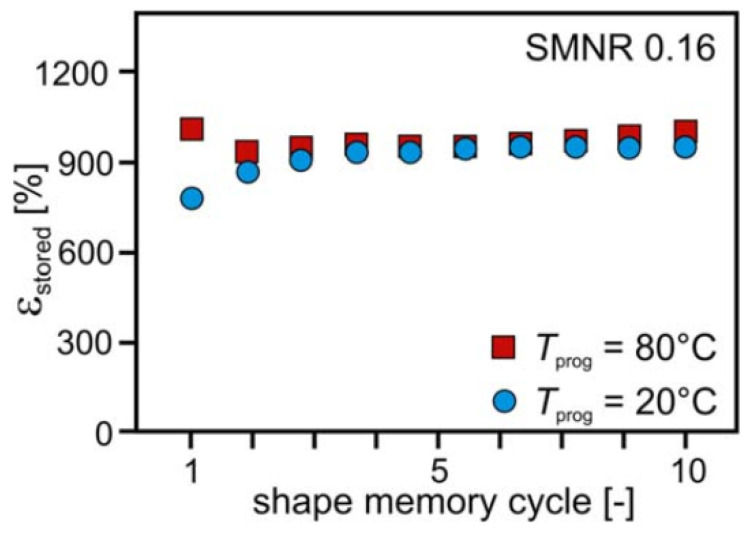
Strain stored for cross-linked SMNR at different programmable temperatures. (Reproduced with permission from [57], Wiley and Sons, 2016).

SMNR is capable of storing a high amount of strain. For an SMNR with a cross-link density of 0.12%, the strain stored was found to be 990%. Figure 3 shows the strain stored (ε_stored_) during a 10 shape memory cycle at 20 and 80 °C, which also indicates the programming temperature slightly affects the storable strain [57,58].

In a study by Chai et al., palmitic acid was used as a swelling agent for shape memory properties. Under a cooling effect, palmitic acid crystallizes onto natural rubber to form a platelet network. This network allows the fabricated shape memory of NR to deform and recover its original shape at room temperature [59].

Blends of epoxidized NR (ENR) with polylactic acid (PLA) and polycaprolactone (PCL) are an example of bio-triggered shape memory polymer. The driving force of recovery is the stored elastic energy of the ENR phase, which is elongated and restricted by the rigid PLA or PCL continuous phase in its temporary shape [60,61].

### 3.3. Synthetic Rubber-Based ShapeMemory Materials

The term synthetic rubber derives from the synthetic analogue to natural rubber, but a great variety of other rubbery materials are produced by chemical synthesis [7].The shape memory properties of synthetic rubbers, such as polyurethane, ethylene propylene diene monomer (EPDM), and silicone rubbers, were largely studied by scientists all over the world. The shape memory effect of zinc-neutralized sulfonated EPDM and fatty acid salts was studied by Weiss and others [62]. Here, ionic aggregates form the cross-linked network and fatty acid salts form the temporary network. The temporary shape was achieved by straining the sample above the melting point of the sample. The next candidates to show the SME are the silicon rubbers. Silicone elastomers were usually blended with paraffin wax or sodium acetate to generate shape memory polymer. Silicon paraffin wax blend showed a one-way shape memory effect, where deformation was achieved by reversible volume expansion/contraction of the wax during melting or crystallization [11,63]. In another study silicone, sodium acetate trihydrate (SAT) blends were prepared. The SAT formed supercooled liquids, where crystallization was obtained by tapping the sample. The deformation is formed at room temperature and the shape can be regained by heating above the melting point of SAT or immersing in water [64].

Polyurethane (PU) after cross-linking, using excess diisocyanate or glycerine, can be used as a shape memory material. The improvement in the increase of the recovery temperature was observed due to the introduction of cross-links. The thermoplastic PU with shape memory effect was analysed by graft polymerizing with the PU backbone [65,66].

### 3.4. Rubber Composites with Carbon-Based Fillers

**Figure 4:**
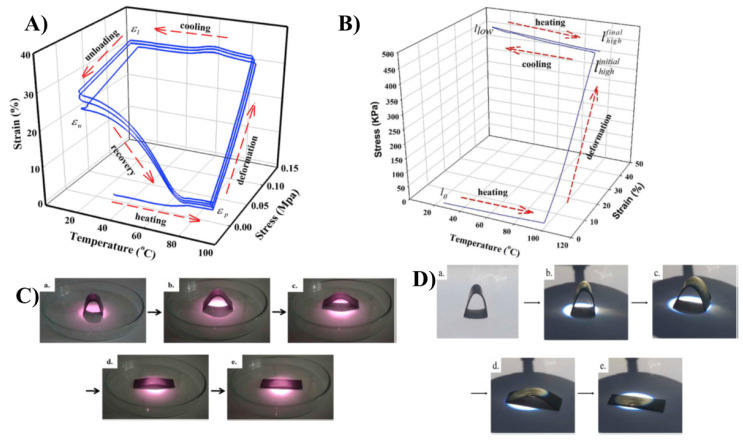
(**A**) One-way shape memory cycle for the natural rubber/paraffin wax 6:4 blend. (**B**) Two-way shape memory cycle for the NR. (**C**). Near-infrared laser-induced shape memory effects of NR/PW 6:4–0.5 CNT [(**a**) original sample, (**b**) irradiated for 15 s, (**c**) 30 s, (**d**) 90 s, and (**e**) 120 s]. (**D**) Sunlight-induced shape memory effects of NR/PW 6:4–0.5 CNT [(**a**) original sample, (**b**) irradiated for 30 s, (**c**) 60 s, (**d**) 90 s, and (**e**) for 120 s] (Reproduced with permission from [67], Elsevier. 2019).

Carbon-based fillers are good thermal conductive fillers. The incorporation of these fillers enhance thermal conductivity and improve the heat distribution between the shape memory device and heat source [67,68,69,70,71]. Carbon nanotubes have a high aspect ratio, which results in high mechanical strength. Fonseca et al. reported that the reduction of the particle size of the filler improves the thermomechanical properties of the material. They have improved the CNT dispersion in thermoplastic polyurethane by functionalization. The carboxylation of CNT established the linkage between CNT and the matrix and improved the thermal diffusivity of the nanocomposite. Reinforcement of CNT into the natural rubber matrix made the composite susceptible to near-infrared laser irradiation, which actsas a trigger to the shape memory process [72]. Lai et al. melted blended natural rubber/paraffin wax/CNT composite and studied the two-way shape memory effect, which involves melt-induced contraction and cooling-induced elongation behaviour. The measurements were conducted using a dynamic mechanic analyser [67].They heated the rubber/paraffin wax/CNT composite to the deformation temperature (Td) of 90 °C at a heating rate of 5 °C/min, then elongated it to the elongation value of 120 kPa, fixed the sample shape, and then cooled it to 10 °C. Then the load was removed, recovery was noted, and the procedure was repeated. Figure 4A shows the one-way shape memory cycle and Figure 4B shows the two-way memory cycle, in which the sample is again heated to a Td of 90 °C, elongated up to 450 kPa, cooled up to 10 °C, and repeated. Figure 4C(a–e) shows the near infrared laser-induced shape memory effects of the NR/paraffin wax/CNT sample and Figure 4D(a–e) shows the images of the sunlight-induced shape memory effects of the NR blend composites. However, applied external stress is needed for the vapour-triggered shape memory process; they overcame this issue by replacing the paraffin wax with beeswax. By adjusting the beeswax composition they have attained the solvent vapour-triggered process [23,31].

**Figure 5:**
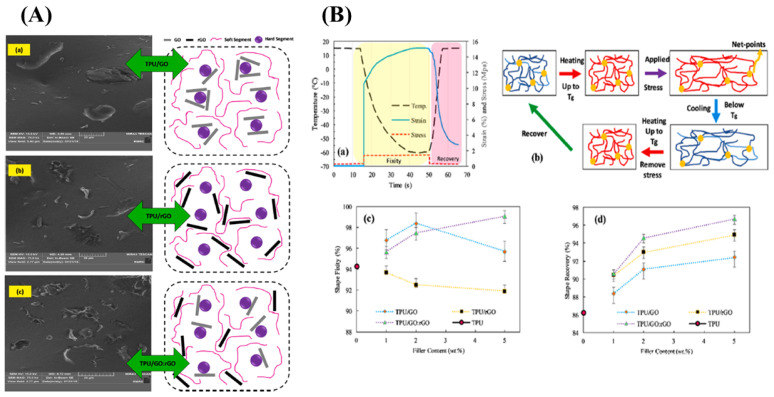
(**A**) Illustrated morphology of the GO-based shape memory material (TPU/GO: rGO) [(**a**) GO platelets, (**b**) rGO platelets, and (**c**) GO:rGO hybrid filler] and the (**B**) molecular mechanism of the shape memory behaviour, shape fixity, and shape recovery [(**a**) Shape memory thermo-mechanical cycle, (**b**) The molecular mechanism of Shape memory behavior (Blue lines: molecular chains with low mobility below Tg; red lines: molecular chains with high mobility above Tg), (**c**) shape fixity and (**d**) shape recovery on neat TPU, TPU/GO, TPU/rGO and hybrid TPU/GO:rGO composites with 1, 2 and 5 wt% filler content] (Reproduced with permission from [30], Elsevier, 2019).

Graphene oxide is layered filler that shows high mechanical and thermal properties; when we reduce the surface oxygen group present in the graphene oxide, it becomes electrically conductive, reduced graphene oxide. Sarmadet et al. used the graphene oxide, reduced the graphene oxide (rGO), and functionalized the reduced graphene, as a filler in the polyurethane matrix. The shape memory effect was studied and a 99.1% of shape fixity value and 96.7% shape recovery value for 5 wt% TPU composite, reinforced with GO: rGO hybrid filler, was obtained [30,45]. Figure 5A illustrates the morphology of the GO-based shape memory material [(a) GO platelets, (b) rGO platelets, and (c) GO:rGO hybrid filler] and Figure 5B shows the shape fixity, recovery, and the molecular mechanism of the shape memory behaviour [(a) Shape memory thermo-mechanical cycle, (b) The molecular mechanism of Shape memory behavior (Blue lines: molecular chains with low mobility below Tg; red lines: molecular chains with high mobility above Tg), (c) shape fixity and (d) shape recovery on neat TPU, TPU/GO, TPU/rGO and hybrid TPU/GO:rGO composites with 1, 2 and 5 wt% filler content]

The incorporation of hybrid fillers, such as CNT/GO, CNT/nano clay, and CNT/carbon black, was also explored by different researchers; there are plenty of hybrid filler combinations that need to be studied [29,73,74]. Liu et al. synthesized the graphene oxide/Trans-1,4-polyisoprene (GO/TPI) nanocomposite and improved the mechanical and thermal properties of the composites at 0.9 phr GO composition; they have also studied the effect of temperature on the rate of shape recovery. They find that the rate of shape recovery increases with temperature [75].

### 3.5. Composites with Metal and Metal Oxide Fillers

**Figure 6:**
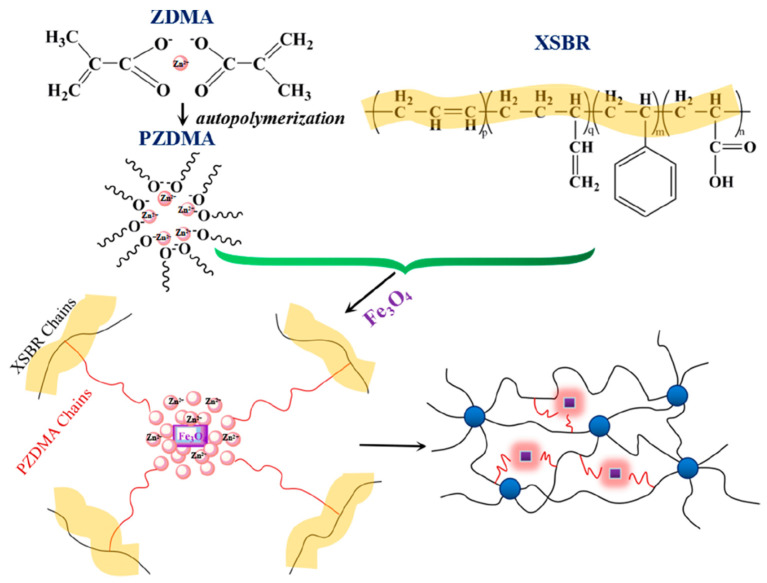
Scheme of the reaction of XSBR, Fe_3_O_4_, and ZDMA (Reproduced with permission from [75], American Chemical Society, 2018).

Magnetically sensitive shapememory materials have major applications in the intelligent biomedical field. Fe_3_O_4_ is a very good nanofiller used for magnetic property, which shows relatively high saturation magnetization, high initial permeability, and low connectivity. The dispersion of the magnetic filler is a major challenge and the in situ addition of magnetic filler could improve the dispersion within the matrix. Via the in situ polymerization reaction, Liu et al. designed a carboxylic styrene-butadiene rubber (XSBR)/ferriferous oxide (Fe_3_O_4_)/zinc dimethacrylate (ZDMA)-based shape memory material with a higher glass transition temperature (20.5 °C), a shape fixation ratio ~100% at room temperature, and a shape recovery ratio of~100%. Figure 6 shows the reaction of XSBR, Fe_3_O_4_, and ZDMA [76].

**Figure 7:**
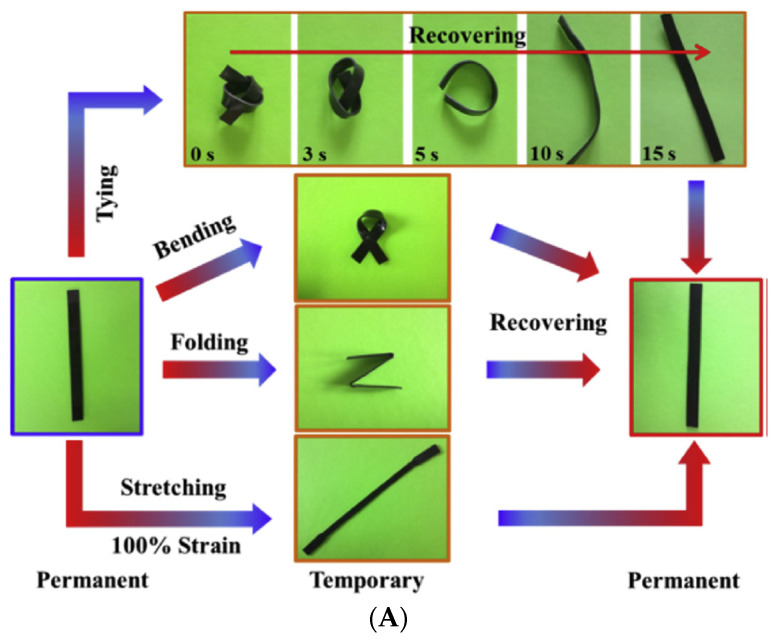

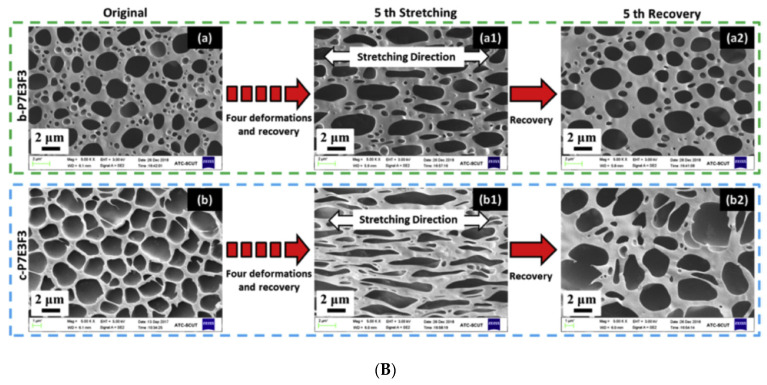
(**A**) Photographs of the shape recovery process of the PLA/ENR/Fe_3_O_4_ TPVs. (**B**) Scanned electron microscopy images of solvent-etched PLA/ENR/Fe_3_O_4_ TPVs (Reproduced with permission from [76], Elsevier, 2019).

Huang et al. successfully synthesized a super tough and locally thermal/magnetic/light-triggered shape memory material with the highest ***R_f_*** (~99%) and ***R_r_*** (>90%) value using polylactide/epoxidized natural rubber thermoplastic vulcanizates by regulating the composition of ferriferous oxide (Fe_3_O_4_), using the dynamic vulcanization method. Figure 7A shows the recovery process, digitally photographed for a better understanding of the process. Figure 7B are the DCM-etched scanning electron microscopy images of the composite, which show very good recovery and have a potential application in intelligent biomedical areas [77].

### 3.6. Composites with Silicon-Based Fillers

The shapememory performance of polyurethane in very low temperatures is significant for automobile industrial uses, such as low spin-out resistance, winter tires, and apparel constituents for usage in exceptionally icy environments, such as locations high above sea level [22,64,78,79,80,81].

By maintaining the sample at a continual elevated temperature or putting on an uninterrupted temperature increase until the strain is entirely well again, shape memory polyurethane and its nanocomposites have been broadly investigated for shape recollection routines under stress, in unrestricted or unconstrained settings [82,83,84,85,86,87]. The distinct program design practice has been used in the majority of published work, and the subsequent reactions have been accomplished under stress-free retrieval or unconstrained situations [78,84,88].

**Figure 8:**
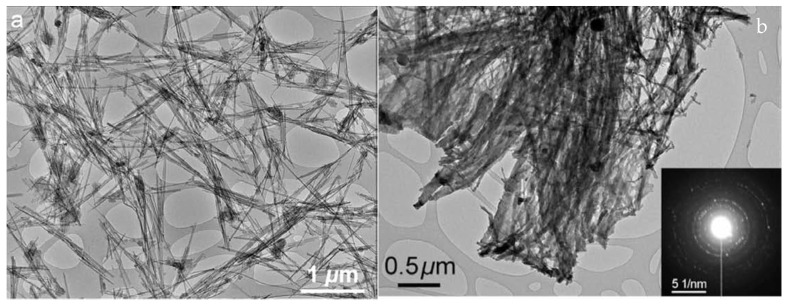
TEM images of untreated (**a**) and heat-treated clay nanopowders (**b**).

Bin Xu et al. studied the attapulgite clay composites with polyurethane and examined the mechanical properties. Comparing the untreated and 850 °C-treated clay showed significant progress in mechanical strength. Heat treatment resulted in the crystallization and formation of layered clay nanoparticles and thereby, the enhancement in mechanical strength. Figure 8 shows the TEM images of untreated and heat-treated silica; it is clear that without treatment it shows a rod-like structure and after treatment the silica forms a bundle structure—it is difficult to break those structures even under ultra-sonication. The SEAD pattern of heat-treated silica and nano-crystallization is also confirmed. In addition, thermal analysis supports the bundle structure of treated silica nano particles [89].

Yang B et al. and Lu H et al. reported the genesis of the indentation size effect (ISE) using a variety of theories, including experimental limitations, work hardening or softening of the objective lens produced during surface groundwork, and structurally core elements of the material, such as work toughening during the production line indentation, elastic restoration from indentation, and the grain consequence of size [90,91,92]. Because of this man-made enhancement, the indention is quite large and the effect is not substantial.

**Figure 9:**
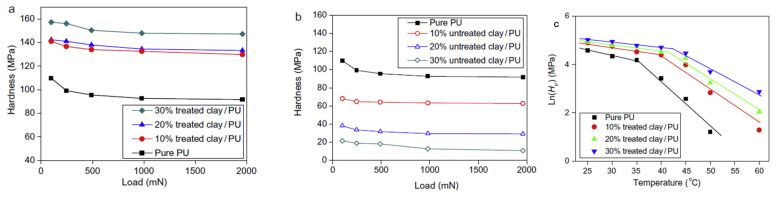
(**a**) Hardness of PU composites with treated clay, (**b**) Hardness of PU composites with untreated clay and (**c**) Microhardness vs. temperature of PU shape memory polymers. (Reproduced with permission from Elsevier, 2009).

Microhardness figures of unadulterated PU, treated, and natural clay-reinforced combinations, as a function of the applied standard load and temperature, are included in Figure 9. The inflexibility of PU-cantered shapememory polymers diminish as the indentation load increases, especially at lower loading [89].

Bin Xu et al. showed that the mechanical properties of the polymer are very much dependent on the pre-treatment of nanofillers. Moisture content in untreated clay acts as a plasticizer and it has a key role in determining the mechanical goods. According to the direction of the work, 4% moisture content can result in an 85% reduction in hardness. Commonly, the glass transition temperature and strength of raw PU–clay nanocomposites decreased. The shape-regaining was comparable across composites based on PU, together with 30 wt% treated clay nanoparticles and unadulterated PU–clay composites, albeit recovery time was slightly slower [89].

G.Tsukada et al., C.E. Friedman et al., and C. Liu et al. identified TPI (trans-1,4-polyisoprene) as a novel category of man-made rubber with shape memory. Its furthermost notable features are an effortlessly adjustable changeover temperature (Tt) at standard temperature and a deformity restoration of more than 200% [9,93,94].

For engineering dynamic polymer composites, controlling the interaction amongst the filler and matrix surfaces is critical. Liu J et al. mediated the charge attraction to create a modified core (silica)–shell (graphene oxide) hybrid, which was then introduced as neoteric filler to a trans-1,4-polyisoprene polymer matrix to create an innovative silica–graphene oxide/trans-1,4-polyisoprene nanocomposite. Fracture toughness, mechanical strength, and heat resistance of the nanocomposites were all increased by hybrid inclusion. They used meticulous investigation from a micro scale to macro scale for analysing and parsing the thermomechanical as well as the shape recall properties of nanocomposites [95].

**Figure 10:**
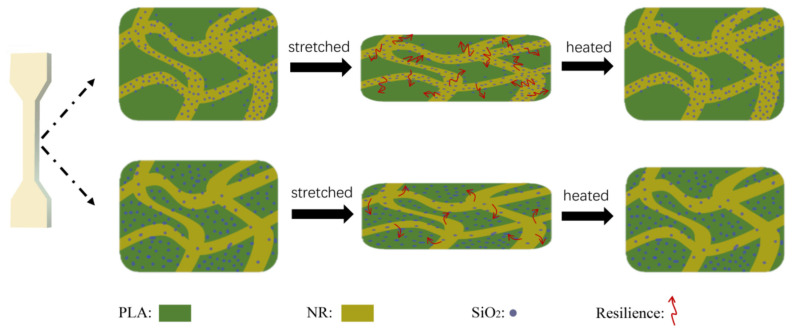
The shape memory behaviour of two types of TPVs. (Reproduced with permission from [82]), Elsevier, 2020).

Yutong Liu et al. used co-continuous polylactide (PLA)/natural rubber (NR)/silica (SiO_2_) TPVs to create a shape memory polymer (SMP). The consequence of the thermodynamic aspect and processing approaches on the selective dispersal of SiO_2_ nanoparticles, as well as the impacts on shape memory behaviour, were investigated. Findings showed that both form fixation and recovery were aided by the co-continuous arrangement. The PLA phase acted as a “button” to control the shape memory actions, even though the distortion of the continuous rubber linkage put in storage enough elasticity to drive the shape and successfully regain course. The fact that tap and bop have distinct shape recovery ratios could be attributed to the careful distribution of silica nanoparticles in TPVs. For taps, the majority of SiO_2_ nanoparticles are located in the NR segment, whereas for bops, a considerable number of silica nanoparticles were also distributed in the PLA phase. The difference between the two types of TPVs in the form of recovery progression is exemplified in Figure 10. The presence of SiO_2_ nanoparticles in the rubber phase has a clear impact on rubber tensile strength, affecting the shape retrieval percentage of TPVs as a result [83].

Abrisham et al. used the heat-actuated approach to study the form remembrance capabilities of polymer composites; the multi-walled carbon nanotubes were used to create the composites. Nanocomposites of thermoplastic polyurethane made of carbon nanotubes (CNT), montmorillonite clay (MMT), and hybrid CNT: MMT nanoparticles were investigated. Mahbod-Abrisham et al. found out that mechanical and shape recall characteristics are enhanced as a result of the appropriate distribution of nanoparticles, in addition to the formation of a greater interfacial area sandwiched between the filler and the matrix, due to the synchronized mixing of CNT and MMT with the matrix [96]. Filler content, crystallinity, and filler localization are all highly influenced. Due to the votive character of MMT nanosheets in improving the distribution of CNT units, hybrid composites have the best heat-actuated shape memory performance. TPU/CNT: MMT-3 sample (96.2%) had the best shape recovery percentage, whereas TPU/CNT: MMT-5 had the highest shape fixity value (99.1%). The synergistic impact of simultaneously including carbon- and silica-based filler into the polymer matrix, which is derived from the marked spreading of hybrid nanoparticles in hybrid composites, was discovered by the research of Mahbod-Abrisham et al. Finally, this synergistic nature paves the way to exceptional thermomechanical behaviour, which is the outcome of hybrid nanoparticles exerting themselves without any alteration. Table 2 shows the shape retention values of the prepared composites.

### 3.7. Composites with Biopolymers and Other Biomaterials

Another advantage of employing SMPs is that biodegradability can be included in the polymer if the therapeutic device is not meant to be everlasting [97,98]. One application of this biodegradable material could be scaffolding devices to aid with bone and tissue restoration. The SMP’s medical potential was recently proved through the use of a self-tightening knot [99]. Biomaterials, such as polylactide (PLA), polyglycolide (PGA), and their copolymer polylactide-co-glycolide (PLGA), have seen increased use in healthcare engineering, such as in environments for targeted drug supply [100,101], medication as a substance for bone implantations and bone fixation measures [102,103,104,105], surgical stitches [106,107], and anastomotic equipment, due to their low cost. This shape memory polymer (SMP) class also includes low crystallinity semicrystalline homopolymers or melt-miscible polymer blends, with at least one of the semicrystalline constituents that are congruent in the molten and amorphous regimes, and at least one semicrystalline fraction [9]. The crystals act as physical cross associations (or harddomains) in this structure, while the composition reliant T_g_ of the unstructured area acts as the conversion temperature.

Within the last decade, the emergence of decomposable implantation components and minimally invasive medical procedures has significantly upgraded the health care sector. Andreas Lendlein and Robert Langer’s work, published in the *Science* journal, describes a class of degradable thermoplastic polymers that can change shape in response to temperature changes. Bulky implants can be put in the body through tiny incisions, and sophisticated mechanical displacements can be performed, automatically attributable to their shapememory capability. To demonstrate the potential of these shape memory thermoplastics in biomedical applications, a smart degradable suture was developed [99].

Liu C et al. investigated two blends, namely polyvinyl acetate (PVAc)/Polylactic acid (PLA) and PVAc/PMMA with poly-vinylidene fluoride(PVDF), which are miscible systems in all blend proportions. PLA and PVDF both have partial crystal-like properties with a crystallinity value of roughly 50%. Crystallinity in the blends is in the 0% to 50% range, depending on the composition proportion, with crystals acting as physical cross-links and crystallinity influencing the modulus of elasticity [9]. The T_g_ of the non-crystalline section serves as a conversion temperature between the two homopolymers and can be adjusted. A copolymer of PLA and poly(glycolide-co-caprolactone), as well as PLA–HA composites, have recently been produced to display favourable shape memory capabilities [108,109].

Water-loving oligomers can be employed to make manifold block copolymers with shape retention capabilities, due to the variety of soft domains available. Wetness can be employed to plasticize the soft realm and drop its T_g_ lower than the contexture temperature to activate form retrieval in these materials, in addition to heat-triggered shape recovery [64,91].

**Figure 11:**
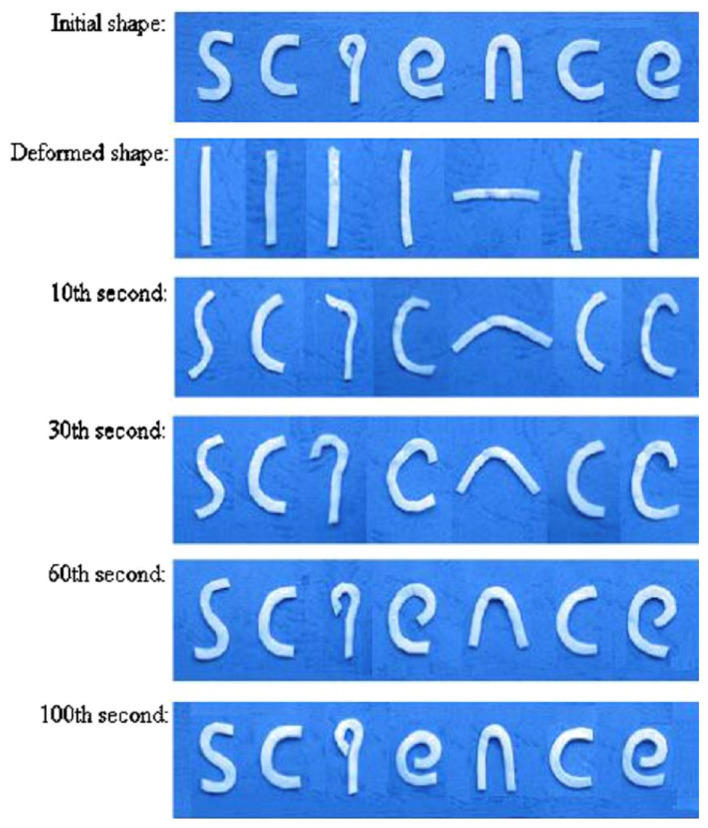
The photographs were taken with a digital camera to depict the progression of shape recovery of the term “science” composed of PDLLA/HA composite. (Reproduced with permission from [107], Elsevier, 2006).

Compounds of Poly(D,L-lactide) (PDLLA) and Hydroxyapatite (HA) with biodegradation, biocompatibility, and shape retention capabilities are technologically advanced. Researchers have looked into some unusual shape memory behaviours. The outcomes reveal that using the experiment methodologies, HA grains have a more dispersed morphology, and PDLLA/HA composites with a specific composite proportion have a significantly superior shape memory end product than crude PDLLA polymer (Figure 11). This suggests that HA entities can increase shape memory and that PDLLA/HA composites have biological uses [109].

In cellulose nanowhisker(CNW)/thermoplastic polyurethane (TPU) nanocomposites, a new strategy for achieving a fast switchable water-sensitive shapememory effect has been demonstrated by Yong Zhu and others [110]. The ability to adapt chemically and mechanically to a cellulose whisker percolation network and the entropicelastomer’s elasticity is the foundation for achieving shape fixity. Yong Zhu et al. also demonstrated the transient deformation and shape recovery in a dry state; when wet, it returns to its former shape [111]. As a result of this phenomenon, the chemo-mechanical relationship and adjustability of the elastomer’s whisker percolation network matrix, provides a new variant of shape memory that can be switched on and off in real-time.

Spider silk is a type of natural shape memory biopolymer that is sensitive to water, allowing it to adapt to a variety of spider demands. Following that, an organizational example of spider silks has been developed, which accounts for a variety of phenomena, including adjustable mechanical characteristics, the starting point, purposes, and ground state of super contraction [53,112,113,114], all related to shape memory.

**Figure 12:**
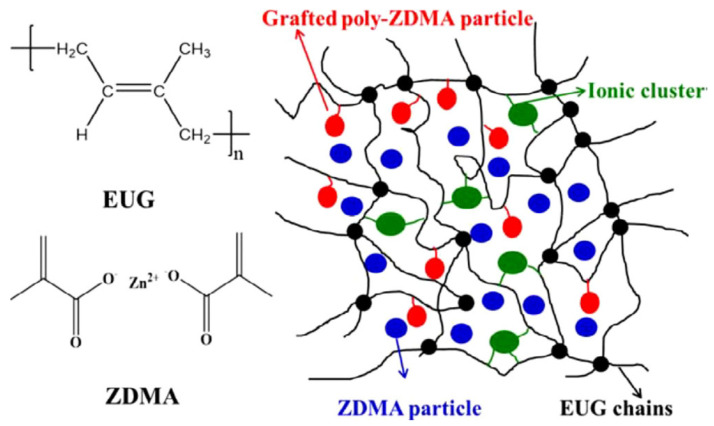
The interaction of EUG chains with ZDMA particles is depicted in this illustration. (Reproduced with permission from [113], Wiley, 2020).

Shape memory polymers manufactured from Eucommiaulmoides gum (EUG) have the potential to be used in biomedical machinery and sensors due to the gum’s renewability, functionality, and biocompatibility. Heat responsive shape memory composites with zinc dimethacrylate(ZDMA) reinforcements were created in the study done by Hailan Kang and colleagues [115]. Figure 12 illustrates ZDMA monomers that were polymerized in situ and evenly distributed in EUG, demonstrating significant interfacial contacts. The shoring up of poly-ZDMA particles is responsible for the significantly enhanced tensile strength and storage modulus in rubber form. Changing the dicumyl peroxide and ZDMA loading changed the interchanging temperature to 29 °C for EUG/ZDMA composites from 50 °C. The EUG/ZDMA composites had extraordinary shape firmness of 95% and a great shape retrieval of 90%, making them ideal for biomedical applications.

## 4. Conclusions

The polymer classification is that elastomers have many superior properties. In addition to these intrinsic properties, an interesting behaviour, called the shape memory effect, is also widely studied. The property of a material, by virtue of whether it can memorize or recover the shape at a particular temperature, is generally termed as the shapememory effect. In polymers, this happens due to the rearrangement of polymer chains. The two parameters—shape fixity ratio and shape recovery rate—are analysed to explain the SME. The addition of fillers, the modification of polymer chains, and the inclusion of the second polymer in the matrix will affect the parameters of SME. The shape memory composites of natural as well as synthetic rubbers, such as SBR, can be produced from fillers of different kinds. The carbon-based fillers, such as graphene oxide, rGO, and CNTs, and inorganic fillers, such as silica, iron oxide nanoparticles, etc., have great potential to enhance shape fixity and recovery. This review examines the factors affecting SME, the different fields of applications, and the future scope of its effects. SME is widely employed in smart devices, such as actuators in robotics. They have been exploited in the biomedical field for artificial implants. Shape memory elastomers are believed to have great potential in biomedical research. Shape memory polyurethanes and composites with biocompatibility are promising materials. The interactions of elastomer chains with the second phase determine the behaviour of the resulting materials. The different possible applications were also mentioned in this article.

## Figures and Tables

**Table 1:** Components to be considered when fabricating SME natural rubber.

Components	Phr
Natural rubber	100
Sulphur	0.2
ZnO	0.15
Zinc diethyldithiocarbamate	0.15

**Table 2:** Shape memory performance of the composites (Reproduced with permission from Elsevier, 2020).

Sample	Shape Recovery (%)	Shape Fixity (%)	Recovery Rate (s)
TPU	86.2	94.3	15.4
1 wt%			
TPU/CNT-1	92.1	95.0	12.8
TPU/MMT-1	90.0	93.6	14.1
TPU/CNT:MMT-1	93.3	93.5	12.4
3 wt%			
TPU/CNT-3	94.7	98.3	12.1
TPU/MMT-3	91.3	94.6	13.7
TPU/CNT:MMT-3	96.2	97.8	11.5
5 wt%			
TPU/CNT-5	92.0	98.5	12.2
TPU/MMT-5	91.0	95.5	14.2
TPU/CNT:MMT-5	95.6	99.1	11.9
